# Data on TGA of precursors and SEM of reduced Cu/ZnO catalysts co-modified with aluminium and gallium for methanol synthesis

**DOI:** 10.1016/j.dib.2019.104010

**Published:** 2019-05-16

**Authors:** R. Guil-López, N. Mota, J. Llorente, E. Millán, B. Pawelec, R. García, R.M. Navarro, J.L.G. Fierro

**Affiliations:** Instituto de Catálisis y Petroleoquímica (CSIC), C/ Marie Curie 2, Cantoblanco 28049, Madrid, Spain

**Keywords:** Methanol synthesis, Copper, Zinc, Gallium, Catalysts

## Abstract

The modification of Cu–Zn catalysts with low amount of Al and Ga (Al+Ga = 3%) was investigated and data corresponding to its influence on the decomposition of the calcined precursors and on the nanomorphology and surface concentration of reduced catalysts were presented in this contribution. The data presented here are supplementary material of the catalysts presented in the *research article “Structure and activity of Cu/ZnO catalysts co-modified with aluminium and gallium for methanol synthesis” published in Catalysis Today* [1]*.*

**Table:** Specifications Table

Subject area	*Chemical Engineering*
More specific subject area	*Catalysis*
Type of data	*Images and figures*
How data was acquired	*Scanning Electron Microscope with energy-dispersive X-ray spectroscopy (Hitachi TM-1000)* *Thermo balance with* heat flow (DSC) and weight changes (TGA) *(Mettler Toledo 85e3)*
Data format	*Analysed*
Experimental factors	*Calcined precursors of catalysts were obtained* by thermal treatment in air of hydroxycarbonate precipitates. The reduced catalysts were obtained from reduction of calcined precursors under a mixture of H_2_/Ar
Experimental features	Thermal decomposition of calcined precursors was studied by thermogravimetry analysing the gas produced by mass spectrometry. The nanomorphology and surface composition of the reduced catalysts were obtained by Scanning Electron Microscopy with energy-dispersive X-ray spectroscopy.
Data source location	*Sustainable energy and chemistry Group, Institute of Catalysis and Petrochemistry (CSIC) Madrid, Spain*
Data accessibility	*Data are provided in this article*
Related research article	*Associated to the research article “Structure and activity of Cu/ZnO catalysts co-modified with aluminium and gallium for methanol synthesis” in Catalysis Today*[1]

**Table:** 

**Value of the data** • The data corresponding to the thermal decomposition of calcined CuO/ZnO precursors indicated that the co-modification with low amount of Al and Ga (Al+Ga = 3%) influences on their carbonate retentionSEM data show that there are not significant differences in the structuration and agglomeration of Cu/ZnO reduced catalyst particles with the modification with Al and Ga. • The energy-dispersive X-ray spectroscopy on reduced Cu/ZnO catalysts revealed the homogeneous composition of the catalyst particles modified with Al and Ga.

## 1 Data

**Fig. 1:**
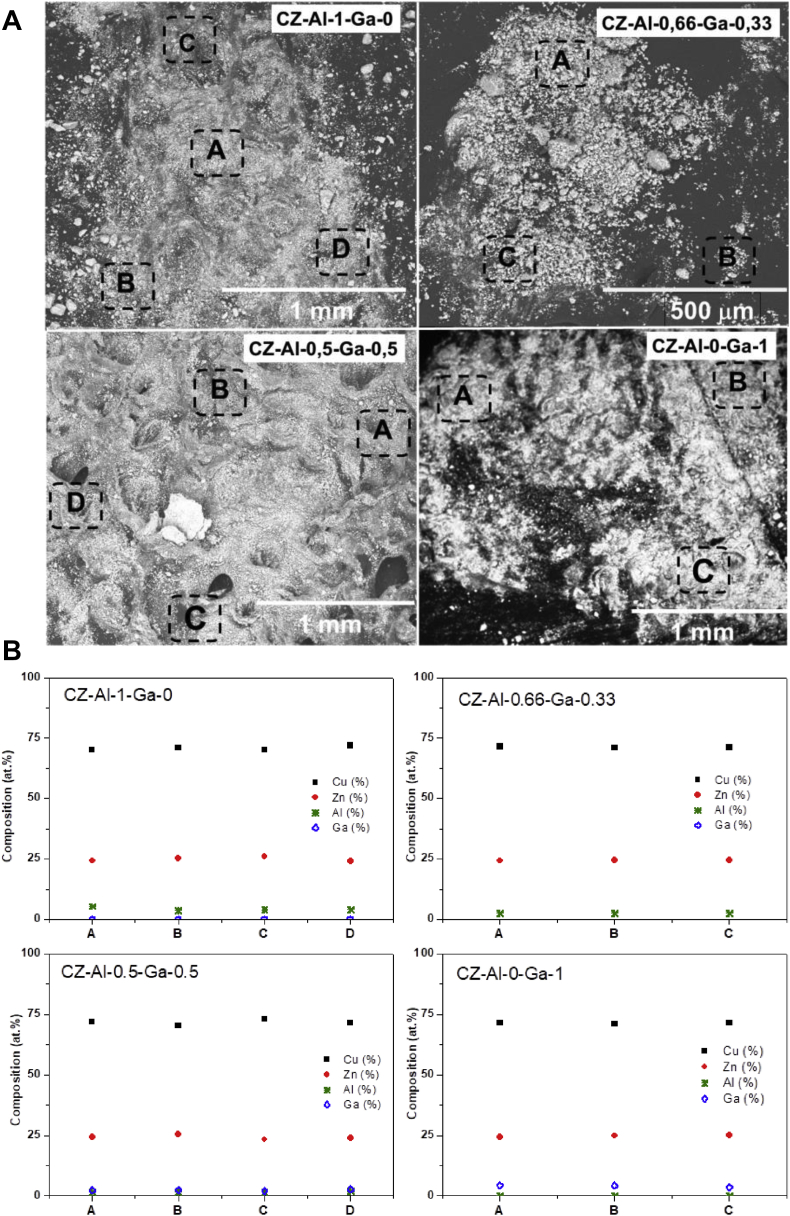
SEM (a) and EDX composition (atomic %) (b) on different areas of CZ-Al-xGa catalysts: CZ-Al-1-Ga-0, CZ-Al-0.66-Ga-0.33 CZ-Al-0.5-Ga-0.5 and CZ-Al-0-Ga-1.

Morphological changes of reduced Cu/ZnO catalysts modified with Al and Ga were analysed by SEM (Fig. 1). Reduced catalysts show similar irregular particles irrespective of the Al and Ga modification. Surface analysis by EDX of reduced Cu/ZnO catalysts modified with Al and Ga (Fig. 1) revealed the homogeneous composition in all analysed particles of catalysts.

**Fig. 2:**
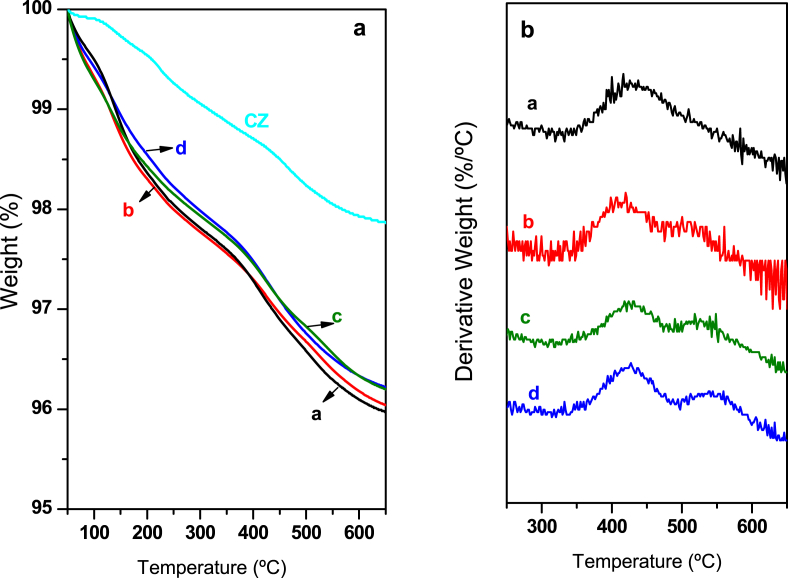
TGA (a) and DTA (b) corresponding to the thermal decomposition of calcined precursors: (a) CZ-Al-1-Ga-0, (b) CZ-Al-0.66-Ga-0.33 (c) CZ-Al-0.5-Ga-0.5 and (d) CZ-Al-0-Ga-1.

The carbonate retention on calcined precursors was analysed by TGA-MS (Fig. 2). The precursors co-modified with Al and Ga show higher concentration of carbonates which decompose at temperatures higher than 500 °C. These carbonates improve the activity and stability of the catalysts obtained after reduction of the calcined precursors.

## 2 Experimental design, materials, and methods

The calcined precursors were prepared by coprecipitation of metal nitrates solutions with sodium or ammonium carbonate to generate mixed hydroxycarbonates [2] followed by calcination in air at 340 °C. Reduced catalysts were obtained by reduction of calcined precursors under diluted hydrogen flow (2.21 vol % H2) at 200 °C (see Table 1).

**Table 1:** Composition of calcined precursors.

Nomenclature	wt %			
	CuO	ZnO	Ga_2_O_3_	Al_2_O_3_
CZ-Al-1-Ga-0	68.2	29.9	0.0	1.9
CZ-Al-0.66-Ga-0.33	67.8	29.7	1.3	1.2
CZ-Al-0.5-Ga-0.5	67.6	29.6	1.0	1.8
CZ-Al-0-Ga-1	67.1	29.4	3.5	0.0

The morphology of the particles in the reduced catalysts was studied by Scanning Electron Microscopy (Hitachi TM-1000). Surface analysis was performed by energy-dispersive X-ray spectroscopy.

Thermal decomposition of calcined precursors was studied by thermogravimetry (Mettler Toledo TGA/SDTA 851e). Decomposition was performed under 25 mL/min of a mixture of O_2_ (20 vol %)/Ar from 40 °C to 600 °C (heating ramp = 10 °C/min). The gas products during decomposition (H_2_O, CO, CO_2_) were analysed by mass spectrometry (Baltzer Prisma QMS 200 TM) quadrupole mass spectrometer.
