# Supplementary material for: Data on TGA of precursors and SEM of reduced Cu/ZnO catalysts co-modified with aluminium and gallium for methanol synthesis
Source: Data Brief. 2019 May 16;24:104010. doi: 10.1016/j.dib.2019.104010 (PMC6538956; doi:10.1016/j.dib.2019.104010)
Supplement: Multimedia component 1 [file mmc1.doc]

Conflict of Interest and Authorship Conformation Form

Please check the following as appropriate:

- All authors have participated in (a) conception and design, or analysis and interpretation of the data; (b) drafting the article or revising it critically for important intellectual content; and (c) approval of the final version.
- This manuscript has not been submitted to, nor is under review at, another journal or other publishing venue.
- The authors have no affiliation with any organization with a direct or indirect financial interest in the subject matter discussed in the manuscript
- The following authors have affiliations with organizations with direct or indirect financial interest in the subject matter discussed in the manuscript:

Author’s name Affiliation

Rufino M. Navarro Instituto de Catalisis y Petroleoquímica (CSIC)

Rut Guil Instituto de Catalisis y Petroleoquímica (CSIC)

Noelia Mota Instituto de Catalisis y Petroleoquímica (CSIC)

Jorge Llorente Instituto de Catalisis y Petroleoquímica (CSIC)

Elena Millan Instituto de Catalisis y Petroleoquímica (CSIC)

Barbara García Instituto de Catalisis y Petroleoquímica (CSIC)

J.L:G. Fierro Instituto de Catalisis y Petroleoquímica (CSIC)
